# Coexistence of Takayasu's Arteritis in Patients with Inflammatory Bowel Diseases

**DOI:** 10.1155/2021/8831867

**Published:** 2021-02-12

**Authors:** Camilla de Almeida Martins, Ana Elisa Rabe Caon, Carolina Bortolozzo Graciolli Facanali, Carlos Walter Sobrado, Sergio Carlos Nahas, Rosa Maria Rodrigues Pereira, Reuma Margalit-Yehuda, Uri Kopylov, Natalia Sousa Freitas Queiroz

**Affiliations:** ^1^Department of Gastroenterology and Division of Colorectal Surgery, University of Sao Paulo School of Medicine, São Paulo, Brazil; ^2^Department of Rheumatology, University of Sao Paulo School of Medicine, São Paulo, Brazil; ^3^Gastroenterology Institute, Sheba Medical Center Tel Hashomer, Sackler School of Medicine, Tel-Aviv University, Tel Aviv, Israel

## Abstract

**Background:**

Takayasu's arteritis (TA) and inflammatory bowel disease (IBD) are chronic inflammatory granulomatous disorders that have rarely been concomitantly reported in case reports and small case series.

**Objective:**

We report a series of seven cases of TA and IBD association in two referral centers with a comprehensive review of literature.

**Methods:**

We analyzed retrospectively the electronic medical charts of TA-IBD patients at the University Hospital of São Paulo, Brazil, and at the Sheba Medical Center at Tel Aviv University, Israel.

**Results:**

Overall, five patients had Crohn's disease (DC) and two had ulcerative colitis (UC), and they were mostly female and non-Asian. All patients developed IBD first and, subsequently, TA. Two underwent colectomy and one ileocecectomy due to IBD activity, while three required cardiovascular surgery due to TA activity. Most patients are currently in clinical remission of both diseases with conventional drug treatment.

**Conclusion:**

Although the coexistence of TA and IBD is uncommon, both seem to be strongly associated through pathophysiological pathways.

## 1. Introduction

Inflammatory bowel diseases (IBD) and Takayasu's arteritis (TA) are chronic inflammatory granulomatous disorders of unknown etiology [1].

TA is a chronic vasculitis of the aorta and its major branches. The inflammation of the involved arteries can lead to stenosis, occlusions, dilatations, and/or aneurysms [2]. It affects especially young females. The clinical manifestations are typically pain, claudication, bruits, absent or diminished pulses, hypertension, and some systemic symptoms, including fever, malaise, weight loss, arthralgia, and myalgia [3]. One important classification based on angiographic findings is described in Table 1 [4, 5].

TA has rarely been reported in patients with IBD. The chance of both diseases occurring in the same patient has been estimated at 1 in 10 billion individuals [6]. Approximately 150 cases of coexistence of these two diseases have been reported in literature since 1976 [7–9], suggesting that there might be a pathophysiological association between these two conditions. In most cases where this association is described, TA occurs after the diagnosis of IBD, within a time frame of 10 to 36 months, and maintains a positive correlation with the activity of intestinal disease.

This study is aimed at reporting a series of seven cases of TA and IBD association in two referral centers. Data regarding clinical features, the temporal relationships between both diagnoses, the relationship with intestinal disease activity, and clinical outcomes following therapeutic interventions will be described in detail. Furthermore, we performed a comprehensive review of literature on the association of TA and IBD.

## 2. Materials and Methods

We collected seven cases from two referral centers: four cases from the Department of Gastroenterology, University Hospital of São Paulo School of Medicine, Brazil, and three from the Department of Gastroenterology, Sheba Medical Center at Tel Aviv University, Israel.

Patients were identified through electronic medical record searches including the keywords “Takayasu,” “arteritis,” and “vasculitis” from both IBD centers, a pool of 5,601 patients with IBD, comprising 3,194 Crohn's disease (CD) patients and 2,407 ulcerative colitis (UC) patients. Data regarding patients' characteristics, disease phenotype, and treatment were retrospectively collected from identified patients using a standardized chart review.

An IBD diagnosis was confirmed by clinical manifestations, endoscopic or surgical features, radiological aspects, and/or standard histological criteria for all included patients. TA diagnosis was based on suggestive clinical findings and confirmed by imaging or histology. Although, it has some limitations in clinical practice, the American College of Rheumatology classification criteria were used in all patients as described in Table 2 [10].

## 3. Results

Overall, seven patients were identified with both TA and IBD. Five patients had CD and two had UC. Four were female. The mean age was 36.6 years old (ranging from 20 to 64). None of them had Asian ancestry. Concerning body weight, one patient was classified as class II obesity, two as overweight, and the others as eutrophic.

Four CD patients showed penetrating behavior and one inflammatory behavior. With respect to CD location, four had ileocolonic involvement and one colonic involvement. Two patients had perianal disease. Two patients with penetrating phenotype were submitted to hemicolectomy, and one was submitted to ileocecectomy. One UC patient was classified as having pancolitis and the other one as having proctitis.

At the time of IBD diagnosis, the main symptoms were bloody diarrhea, abdominal pain, and weight loss. The most common TA symptoms were headache, fatigue, upper limb claudication, weight loss, fever, arthralgia, and syncope. Two of seven cases had extraintestinal manifestations, such as arthritis and ankylosing spondylitis. One of them had osteoporosis, and the other had hypothyroidism.

All patients developed IBD first and, subsequently, TA. The mean age at diagnosis was 21.9 years (ranging from 11 to 30) for IBD diagnosis and 26.6 years for TA. The time frame between both diagnoses varied from 4 months to 10 years. The mean duration of follow-up is 11.5 years (ranging from 1 to 39 years). Regarding Hata classification, two patients had characteristics of type V, one patient type I, one patient type IIa, one patient type IIb, one patient type III, and another patient type IV. The most important findings are described in Table 2.

The seven patients used the following IBD treatments: two were medically treated with combination therapy (infliximab and azathioprine), one used infliximab monotherapy, two azathioprine and oral 5-aminosalicylic acid, one azathioprine alone, and another one oral 5-aminosalicylic acid alone. They are currently in clinical remission.

With regard to TA therapy, two patients required a cardiovascular surgery due to an ascending aortic aneurysm associated with aortic insufficiency. These patients had surgical correction of aneurysm and mechanical valve replacement. Another patient also had a vascular procedure. She presented with a contained rupture in the descending aorta at the TA diagnosis and underwent an urgent endovascular repair surgery of the thoracic aorta. As an initial therapy, she used corticosteroids and cyclophosphamide, but due to an exacerbation, she subsequently changed to tocilizumab and methotrexate, with partial response. Now she is a candidate for the infliximab therapy. Further details on TA therapy are being described in Table 3.

## 4. Discussion

Even though the coexistence of IBD and TA in the same individual is uncommon, there are approximately 150 cases of this association reported in literature [8, 9, 11, 12].

In 2009, Konopka et al. described the first case in Brazil of concurrent IBD and TA in a Caucasian woman with CD manifesting with fever, blood pressure difference between the upper limbs, and subclavian and abdominal bruits [13]. Computed tomography angiography confirmed the diagnosis of TA.

Reny et al. calculated the expected prevalence of CD in patients with TA to be approximately 0.05-0.2%, while the prevalence of IBD in TA cohorts can be as high as 6-9% [9, 14, 15], which is superior to the expected prevalence of IBD in the general population. Sy et al. identified TA as the most frequent vasculitis related to IBD in a multicentric study [8], further reinforcing its coexistence is not incidental.

In the Brazilian IBD center, we have 2,749 patients with IBD, comprising 1,288 CD and 1,461 UC patients. We found a prevalence of TA in this cohort of 0.14%. In the Sheba Medical Center in Israel, there are 2,852 patients with IBD, comprising 1,906 CD and 946 UC patients. The prevalence of TA in this cohort is 0.1%. Similarly, Oshitani et al. showed a prevalence of TA in a Japanese cohort of UC individuals of 0.21% [16]. It is known that TA is more prevalent in Asians [17]. Although none of the individuals have self-reported Asian ancestry, it is important to emphasize that the Brazilian population is diverse and miscegenated, and the Israeli population is mostly composed of Jewish-descent immigrants.

Consistent with literature, most of our patients were female and had TA onset at a younger age in comparison with patients without IBD-associated disease. Comparably, all of our patients were diagnosed with IBD before TA [9, 14, 18].

IBD diagnosis can be challenging in TA patients, as vasculitis can potentially lead to mesenteric ischemia and exhibit confounding symptoms and endoscopic findings [8]. In a Japanese retrospective study that analyzed colonoscopy results of patients previously diagnosed with TA, the major endoscopic finding of IBD-TA at initial diagnosis was discontinuous focal mucosal inflammation [11].

The pathophysiological mechanisms involved in TA-IBD association are uncertain. TA and UC are linked to common HLA types, such as HLA-B∗52.01, which in turn is strongly considered the key HLA type in TA-UC. Akiyama et al. identified the HLA-C 12:02 and DRB01∗15:02 correlation with this coexistence. This study also showed DRB-1∗15:02-DQA-1∗01:03-DQB-1∗06:01-DPB-1∗09:01 to be significantly associated with TA-UC, as it predisposes to both diseases in Japanese population. Non-HLA markers may as well have an implication [11, 14, 19].

In contrast, no HLA genotype has been associated with TA and CD, although both diseases share some similar clinical characteristics. They are common in young women, have granulomatous nature, and show clinical response to treatment with corticosteroids and immunosuppressive drugs. Several factors that play important roles in autoimmunity are present in TA and CD, such as lymphocyte subtype and proinflammatory cytokines (IFN*γ*, TNF*α*, IL-6, IL-17, IL-18) [20–22]. Also, granulomatous vasculitis was evidenced in resected intestine from a patient with TA-CD [23].

As literature suggests, the most common symptoms related to TA identified in our patients were also claudication of extremities, malaise, weight loss, arthralgia, and headache [8, 14, 15]. Syncope is not a frequent symptom reported in TA-IBD association, and it was only in one patient present.

One of our cases had hypothyroidism. Esatoglu et al. investigated associated inflammatory diseases in 198 TA patients. In addition to IBD, they identified 6% of patients with autoimmune thyroid disease, which is consistent with the prevalence in the general population. However, concomitant UC and Hashimoto's thyroiditis might be more common in patients with multiple autoimmune disorders [24].

Most treatments used for IBD are also effective for TA, since both diseases share similar inflammatory pathways. In a recent systematic review, treatments and outcomes of patients with TA-IBD and TA alone were similar [8].

Glucocorticoids are the first-line treatment of TA, the and European League Against Rheumatism (EULAR) recommends it should be given in combination with a nonbiologic glucocorticoid-sparing agent, such as methotrexate, mycophenolate mofetil, leflunomide, azathioprine, or cyclophosphamide. Biological therapy as anti-TNF or tocilizumab may be used in refractory or relapsing disease [2].

Sato et al. described the first case of TA-UC successfully treated with JAK inhibitor tofacitinib. Both diseases were active, and the patient had previously failed to mesalazine, azathioprine, golimumab, and vedolizumab. After therapy initiation, symptoms rapidly improved, inflammatory markers decreased, and enhanced CT scan revealed reduction in wall thickness of involved arteries. Also, patient developed endoscopic remission and no relapses were observed within a 6-month follow-up [12].

All of our patients are in remission to date with conventional treatments for both diseases, and only one was submitted to colectomy before the correct diagnosis. Two patients were submitted to cardiovascular procedures, such as valvar replacement and aorta aneurysm correction surgery. There were no deaths, colorectal dysplasia or neoplasia, strokes, or myocardial infarctions. A Turkish retrospective study including 12 TA-IBD patients with an average follow-up of 8.5 years found that the most common complications were cardiovascular events and perianal fistula [9].

Our study has some limitations to consider. First, it consists of a retrospective and transverse design. Also, it is based in a nonsystematic analysis of medical records. However, all available data was extracted.

## 5. Conclusions

In conclusion, the association of IBD and TA is rare, but the existence of common pathophysiological pathways indicates that these conditions may be strongly associated. Since both diseases can be treated with the same agents, diagnostic delay may occur. Novel therapies for IBD can be useful for concomitant treatment of TA.

## Figures and Tables

**Table 1:** Angiographic classification of Takayasu's arteritis [4].

Type	Vessel involvement
I	Branches from the aortic arch
IIa	Ascending aorta, aortic arch and its branches
IIb	Ascending aorta, aortic arch and its branches, thoracic descending aorta
III	Thoracic descending aorta, abdominal aorta, and/or renal arteries
IV	Abdominal aorta and/or renal arteries
V	Combined features of types IIb and IV

**Table 2:** The 1990 American College of Rheumatology classification criteria for Takayasu's arteritis [10].

Criteria	Definition
Age at disease onset ≤ 40 years	Development of symptoms or findings related to TA at age < 40 years
Claudication of extremities	Development and worsening of fatigue and discomfort in muscles of 1 or more extremity while in use, especially the upper extremities
Decreased brachial artery pulse	Decreased pulsation of 1 or both brachial arteries
Blood pressure difference > 10 mmHg	Difference of >10 mmHg in systolic blood pressure between arms
Bruit over subclavian arteries or aorta	Bruit audible on auscultation over 1 or both subclavian arteries or abdominal aorta
Arteriogram abnormality	Arteriographic narrowing or occlusion of the entire aorta, its primary branches, or large arteries in the proximal upper or lower extremities, not due to arteriosclerosis, fibromuscular dysplasia, or similar causes; changes usually focal or segmental

For purposes of classification, a patient shall be said to have TA if at least 3 of these 6 criteria are present. The presence of any 3 or more criteria yields a sensitivity of 90.5% and a specificity of 97.8%.

**Table 3:** Main clinical characteristics of seven patients with IBD and TA.

Patient	Sex	Age	Age at IBD diagnosis	Age at TA diagnosis	Interval from onset	IBD type	IBD surgeries	IBD drug therapy	TA drug therapy	Vascular procedures
1	F	23	11	14	3 y	UC	—	AZA+5-ASA	Warfarin	Repair of ascending aortic aneurysm with graft and mechanical aortic valve replacement
2	M	20	16	20	4 y	CD	Hemicolectomy	AZA	AAS+PDN+MTX	
3	F	43	30	31	1 y	CD	—	IFX	AAS	
4	F	64	25	31	6 y	CD	Hemicolectomy	IFX+AZA	AAS	
5	F	21	21	21	4 months	UC	—	5-ASA	Csa+PDN; anti-IL-6R+MTX AAS	Repair of descending thoracic aortic aneurysm with graft.
6	M	40	22	31	9 y	CD	—	5-ASA+AZA	PDN	
7	M	45	28	38	10 y	CD	Ileocecectomy	6-MP IFX+AZA	PDN+anti-IL-6R warfarin	Repair of ascending aortic aneurysm with graft and mechanical aortic valve replacement

IBD: inflammatory bowel disease; TA: Takayasu's arteritis; UC: ulcerative colitis; CD: Crohn's disease; AZA: azathioprine; 5-ASA: 5-aminosalicylic acid; IFX: infliximab; AAS: acetylsalicylic acid; MTX: methotrexate; antiIL6R: tocilizumab; 6-MP: 6-mercaptopurine; PDN: prednisone; Csa: cyclophosphamide.

## Data Availability

All data was extracted from medical records. This data was used to support the findings of this study, and they are included in the article.
